# Functional implications of K_V_7 channel phosphorylation

**DOI:** 10.1186/2050-6511-13-S1-A46

**Published:** 2012-09-17

**Authors:** Isabella Salzer, Wei-Quiang Chen, Helmut Kubista, Gert Lubec, Stefan Boehm, Jae-Won Yang

**Affiliations:** 1 Department of Neurophysiology and Neuropharmacology, Center for Physiology and Pharmacology, Medical University of Vienna, 1090 Vienna, Austria; 2 Department of Pediatrics, Medical University of Vienna, 1090 Vienna, Austria; 3 Institute of Pharmacology, Center for Physiology and Pharmacology, Medical University of Vienna, 1090 Vienna, Austria

## Background

The family of K_V_7 potassium channels, particularly K_V_7.2, K_V_7.3, and K_V_7.5 controls neuronal excitability. Numerous neurotransmitters acting via G protein-coupled receptors signaling via Ca^2+^/calmodulin or depletion of membrane phosphatidylinositol-4,5-bisphosphate (PIP_2_) tightly regulate K_V_7 channel function. Moreover, the phosphorylation of K_V_7 channels has been proposed to play a crucial role. However, *in vivo* phosphorylation sites and their functional implications need to be determined.

## Methods

To investigate the role of steady-state K_V_7 channel phosphorylation, superior cervical ganglion (SCG) neurons were pretreated for 30 min with different kinase inhibitors (GW8510: 10 µM, SB415286: 1 µM, SB203580: 10 µM, H7: 10 µM) which block CDK5, GSK3, p38 MAPK, and PKC as well as PKA, respectively. Thereafter, oxotremorine M (OxoM) or bradykinin-induced inhibition of the M-currents (primarily through K_V_7.2/7.3 heterotetramers) was tested.

## Results

Inhibition of CDK5 shifted the concentration-response curve for OxoM to the left, but not that of bradykinin. Similarly, GW8510 treatment of tsA201 cells, heterologously expressing K_V_7.2 channels and M_1_ receptors, caused a leftward shift of the OxoM concentration-response relation. In mass-spectrometric studies, several phosphorylated amino acid residues in the C-terminus of native and heterologously expressed K_V_7.2 channels were detected, 5 of them are located within the putative PIP_2_ binding site. CDK5 was predicted to target serines S427 and S446. In contrast to S446, mutation of S427 to alanine significantly increased K_V_7.2 channel sensitivity towards inhibition via M_1_ receptors. Additionally, treatment with GW8510 failed to cause any further effect. Nevertheless, these alanine mutations did not influence the channel-voltage dependence.

## Conclusions

Hence, phosphorylation of C-terminal serine residue 427 determines K_V_7.2 modulation by M_1_ muscarinic, but not by B_2_ bradykinin receptors, suggesting that the phosphorylation state of S427 regulates the affinity of the K_V_7.2 C-terminus for PIP_2_.

